# Association of Polyphenols from Oranges and Apples with Specific Intestinal Microorganisms in Systemic Lupus Erythematosus Patients

**DOI:** 10.3390/nu7021301

**Published:** 2015-02-16

**Authors:** Adriana Cuervo, Arancha Hevia, Patricia López, Ana Suárez, Borja Sánchez, Abelardo Margolles, Sonia González

**Affiliations:** 1 Department of Functional Biology, University of Oviedo, C/Julián Clavería s/n Oviedo, 33006 Asturias, Spain; E-Mails: cuervoadriana.uo@uniovi.es (A.C.); lopezpatricia@uniovi.es (P.L.); anasua@uniovi.es (A.S.); 2 Department of Microbiology and Biochemistry of Dairy Products, Instituto de Productos Lácteos de Asturias-Consejo Superior de Investigaciones Científicas (IPLA-CSIC), Paseo Río Linares s/n Villaviciosa, 33300 Asturias, Spain; E-Mails: ahevia@ipla.csic.es (A.H.); borja.sanchez@uvigo.es (B.S.); amargolles@ipla.csic.es (A.M.)

**Keywords:** lupus, polyphenols, microbiota, orange, apple, red wine

## Abstract

Our group has recently shown the existence of a gut microbial dysbiosis in systemic lupus erythematosus (SLE), supporting previous evidence involving intestinal bacteria in the initiation and amplification of autoimmune diseases. While several studies have addressed the use of dietary fibres to modify intestinal microbiota, information about other correlated components, such as polyphenols, is scarce. The aim of this work was to identify dietary components able to influence this altered microbiota in 20 SLE women and 20 age-matched controls. Food intake was recorded by means of a food frequency questionnaire. The intake of fibres was calculated from Marlett tables, and Phenol-Explorer was used for polyphenol consumption. Results showed positive associations between flavone intake and *Blautia*, flavanones and *Lactobacillus*, and dihydrochalcones and *Bifidobacterium* in the SLE group. Regarding the controls, dihydroflavonols were directly associated with *Faecalibacterium*, whereas flavonol intake was inversely associated with *Bifidobacterium*. From the food sources of these polyphenols related to microbiota, orange intake was directly associated with *Lactobacillus* and apple with *Bifidobacterium* in SLE, whilst red wine was the best contributor to *Faecalibacterium* variation. The association between common foods and particular microbial genera, reported to be decreased in SLE, could be of great importance for these patients.

## 1. Introduction

Gut microbiota has been related to the modulation of the immune system and to anti-oxidant defence [1,2,3]. We have recently published the existence of a gut microbial dysbiosis in patients with systemic lupus erythematosus (SLE). The decrease in the ratio of *Firmicutes* to *Bacteroidetes* in SLE subjects [4] supports previous evidence involving intestinal bacteria in the initiation and amplification of inflammatory processes and autoimmune diseases [5,6,7]. In this regard, common clinical features associated with SLE, such as intestinal vasculitis, could have an influence on the observed dysbiosis. In relation to this, high *Bacteroidetes* levels have also been found in patients with inflammatory bowel disease [8]. In this context, it has been reported that patients with metabolic syndrome fed with a low fat/high carbohydrate diet suffered an increase in *Bacteroides* spp. and *Bifidobacterium* spp. [9]. Other previous studies have positively associated levels of *Firmicutes* with a low-fat/high-fibre diet [10] and the increase in *Firmicutes/Bacteroidetes* ratio with whole grain supplementation [11]. Also, it has been found that the microbiota of people with a long-term diet rich in animal protein and saturated fat presents more *Bacteroides* [12]. Apart from this, other bioactive compounds from diet, such as polyphenols, are able to modulate the intestinal microbiome [13], and have shown promising results in models of autoimmune diseases [14]. To date, there are few studies which have focused on the interactions between polyphenol intake and microbiota: evidence from animal and human studies has shown that supplementation with polyphenol-rich food, such as red wine [15], tea [13], cocoa [16] or blueberries [17,18], modulates some intestinal bacterial populations, but the results were not conclusive. While Queipo-Ortuño *et al.* have recently published changes in *Proteobacteria*, *Fusobacteria*, *Firmicutes* and *Bacteroidetes* concentrations in humans after dietary intervention with alcoholic beverages (red wine and gin) and de-alcoholized red wine [15], animal studies have found a lower proportion of *Clostridium* and *Lactobacillus* in polyphenol-treated rats with respect to the control [19]. Moreover, the effects of cocoa flavanols remain controversial: an increase in *Lactobacillus* and *Bacteroides* has been described in humans [20], but evidence in animal studies showed a decrease of *Bacteroides*, *Clostridium* and *Staphylococcus* [16].

Thus, as this imbalance is susceptible to being modified by shifting dietary patterns, the overall aim of this paper was the identification of dietary components associated with this altered microbiota. Knowledge of the interactions between diet and microbiota in SLE subjects may be useful in the future to establish some basis for the rational design of functional foods and dietary intervention strategies meant to improve the clinical manifestations of these chronic patients.

## 2. Subjects and Methods

The study sample comprised 20 SLE patients selected from the updated Asturian Register of Lupus [21]. All of them fulfilled at least four of the American College of Rheumatology criteria for SLE [22], were women of Caucasian origin, aged between 35–70 years and had no active disease at the time of sampling (Systemic Lupus Erythematosus Disease Activity Index (SLEDAI) score ≤ 8). Patients were also asked precise questions regarding treatment received during the previous 6 months. Only those individuals who had not used antibiotics, glucocorticoids, immunosuppressive drugs, monoclonal antibodies, or other immunotherapies were recruited for the study. Eighteen patients were receiving antimalarial treatment, and all of them were regular consumers of nonsteroidal anti-inflammatory drugs. Twenty age-matched healthy women from the same population were recruited as controls.

Ethics approval for this study (reference code AGL2010-14952; grant title “Towards a better understanding of gut microbiota functionality in some immune disorders”) was obtained from the Bioethics Committee of CSIC (Consejo Superior de Investigaciones Científicas) and from the Regional Ethics Committee for Clinical Research (Servicio de Salud del Principado de Asturias, nº 13/2010) in compliance with the Declaration of Helsinki. All determinations were performed with fully informed written consent from all participants involved in the study.

### 2.1. Nutritional Assessment

Dietary intake was assessed by means of an annual semi-quantitative food-frequency questionnaire (FFQ) of 160 items, previously validated for polyphenol intake in other samples [23]. The FFQ was organized by groups of food and open-ended, allowing foods consumed by the subject and not registered in the questionnaire to be recorded. During a personal interview, subjects were asked item-by-item whether they usually ate each food (daily, weekly or monthly), and, if so, how much they usually ate. To facilitate the recording of the quantities of food consumed, 3 different serving sizes of each cooked food were presented in pictures to the participants, so that they could choose from up to 7 serving sizes (from “less than the small one” to “more than the large one”). For some of the foods consumed, amounts were recorded in household units, by volume, or by measuring with a ruler. Special attention was paid to cooking practices, number and amount of ingredients used in each recipe, as well as questions concerning menu preparation (e.g., type of oil used, type of milk) and other relevant information for the study, such as the consumption of skin in fruit. Furthermore, variability in the consumption of some foods, especially fruits and vegetables, according to the season was taken into account. The consumption of foods was converted into energy and saturated fatty acid intake using the nutrient Food Composition Tables developed by the *Centro de Enseñanza Superior de Nutrición Humana y Dietética* (CESNID) [24]. Dietary fibre was determined from Marlett Food Composition Tables [25] and animal protein from the USDA Database [26]. The content of flavonoids in the foods consumed in the sample, with the exception of spices and condiments, was calculated using the Phenol-Explorer Database. This database contains detailed information of the polyphenol content in more than 400 foods, mainly determined by high-performance liquid chromatography (HPLC), gas chromatography (GC), and capillary electrophoresis (CE), and is compiled from more than 1300 publications, being at the moment one of the main tools used in human studies [27].

### 2.2. Anthropometric Measures

Body mass index (BMI) was calculated using Quetelet formula. Weight was measured on a scale with an accuracy of ±100 g (Seca, Hamburg, Germany). Height was registered using a stadiometer with an accuracy of ±1 mm (Año-Sayol, Barcelona, Spain). Subjects stood barefoot, in an upright position and with the head positioned in the Frankfort horizontal plane.

### 2.3. Microbiological Analyses

Faecal DNA extraction, 16S rRNA amplification sequencing of 16S rRNA gene-based amplicons and the sequence-based microbiota analysis were reported elsewhere [4]. The raw sequences reported in this article are deposited in the NCBI Short Read Archive (SRA) (study accession number: SRP028162).

### 2.4. Statistical Analysis

Statistical analysis was performed using IBM-SPSS version 19.0 (SPSS-Inc., Chicago). Goodness of fit to normal distribution was analysed with the Kolmogorov-Smirnov test. Since the relative abundance of *Lactobacillus* did not fulfill this criterion, the variable was logarithmically transformed to perform the analysis. For descriptive purposes, mean values were presented on untransformed variables. Multivariate analysis, including age and energy intake, were conducted to examine the difference in the mean intake of animal protein, saturated fatty acids, dietary fibre and flavonoids between SLE patients and controls, while the Student *t*-test was used to evaluate the difference in consumption of the main food sources of flavonoids. In order to investigate the association between the intake of animal protein, saturated fatty acids, dietary fibre and flavonoids and faecal relative abundance of the studied microorganisms, multiple linear regression analysis was carried out. Age and energy intake were also included as covariates in the models. The main food sources of the dietary components previously related to microbiota were selected and placed in a multiple stepwise regression analysis to explore their independent effect. The statistical parameters employed were β (standardized regression coefficient) and *R*^2^ (coefficient of multiple determinations).The conventional probability value for significance (0.05) was used in the interpretation of results. Considering the standard deviation of the microbial relative abundance and the standard deviation of the regression errors, we could reject the null hypothesis with an estimated probability around 0.8 and a type I error probability of 0.05 for the three models (calculated using the software PS: Power and Sample Size Calculation version 3.0.43 (Vanderlbilt University, Nashville, TN, USA)).

## 3. Results

General characteristics and daily intake of dietary components previously associated with microbiota are compared between SLE patients and control subjects in Table 1. Both groups were similar for all the studied variables.

**Table 1:** Characteristics of the evaluated variables in systemic lupus erythematosus (SLE) and controls.

	SLE (*N* = 20)	Control (*N* = 20)	Reference
Age (year)	49.25 ± 10.71	46.95 ± 8.60	[4]
BMI (kg·m^−2^)	26.11 ± 5.31	25.17 ± 4.16	[4]
Energy (kcal·day^−1^) ^a^	2189.65 ± 722.42	1858.53 ± 332.85	[4]
Animal protein (g·day^−1^) ^a,b^	104.70 ± 27.60	100.85 ± 20.86	This work
Saturated fatty acids (g·day^−1^) ^a,b^	25.19 ± 14.09	24.66 ± 6.04	[4]
Dietary fiber (g·day^−1^) ^a,b^	18.06 ± 10.04	20.32 ± 8.52	[4]
Total flavonoids (mg·day^−1^) ^a,b^	400.13 ± 259.94	436.34 ± 189.70	This work
Anthocyanins (mg·day^−1^) ^a,b^	20.81 ± 30.30	29.73 ± 30.65	
Dihydrochalcones (mg·day^−1^) ^a,b^	2.70 ± 4.42	3.26 ± 3.58	
Dihydroflavonols (mg·day^−1^) ^a,b^	0.34 ± 0.80	1.96 ± 2.82 *	
Flavanols (mg·day^−1^) ^a,b^	298.54 ± 222.16	283.47 ± 160.21	
Flavanones (mg·day^−1^) ^a,b^	38.22 ± 29.44	45.31 ± 32.81	
Flavones (mg·day^−1^) ^a,b^	2.48 ± 2.39	3.96 ± 3.92	
Flavonols (mg·day^−1^) ^a,b^	38.00 ± 30.26	52.05 ± 25.55	
Isoflavones (mg·day^−1^) ^a,b^	5.52 ± 16.81	15.92 ± 66.13	

Multivariate analysis adjusted by ^a^ age and ^b^ energy intake; Results are presented as estimated marginal mean ± SD; * *p* ≤ 0.05.

In order to explore the association between dietary components and faecal microbiota with independence of age and energy intake, linear regression analysis was conducted. Animal protein, saturated fatty acids and dietary fibre were not associated with microbial genera in any of the studied groups (Table 2).

**Table 2:** Linear regression analyses for daily intake of animal protein, saturated fatty acids (SFA) and dietary fiber on fecal microbial in patients with systemic lupus erythematosus (SLE) and controls.

	Group	Animal protein (g·day^−1^)		SFA (g·day^−1^)		Dietary fiber (g·day^−1^)	
		*R*^2^	β	*R*^2^	β	*R*^2^	β
***Blautia* (%)**	L	0.129	0.206	0.273	−0.874	0.109	0.129
	C	0.028	−0.117	0.016	0.031	0.046	−0.205
***Clostridium* (%)**	L	0.271	0.431	0.170	0.363	0.140	−0.116
	C	0.116	0.343	0.007	0.026	0.007	−0.022
***Lactobacillus* (%)**	L	0.041	−0.237	0.024	−0.312	0.031	0.225
	C	0.203	−0.347	0.091	−0.030	0.174	0.336
***Lactococcus* (%)**	L	0.345	0.447	0.213	−0.193	0.218	0.150
	C	0.065	−0.210	0.029	−0.086	0.029	−0.085
*Faecalibacterium* (%)	L	0.063	−0.111	0148	0.641	0.086	−0.232
	C	0.143	0.272	0.075	0.028	0.074	0.000
*Bacteroides* (%)	L	0.005	−0.026	0.017	0.232	0.118	0.439
	C	0.125	0.110	0.291	0.483	0.115	−0.045
*Bifidobacterium* (%)	L	0.121	−0.126	0.147	0.402	0.299	0.567
	C	0.323	−0.154	0.302	−0.046	0.371	−0.309

L = SLE (*N* = 20); C = Control (*N* = 20); SFA = Saturated Fatty Acids; Results derived from multiple linear regression analysis including age and energy intake as covariates; *R^2^*: coefficient of multiple determinations; β: standardized regression coefficient.

**Figure 1:**
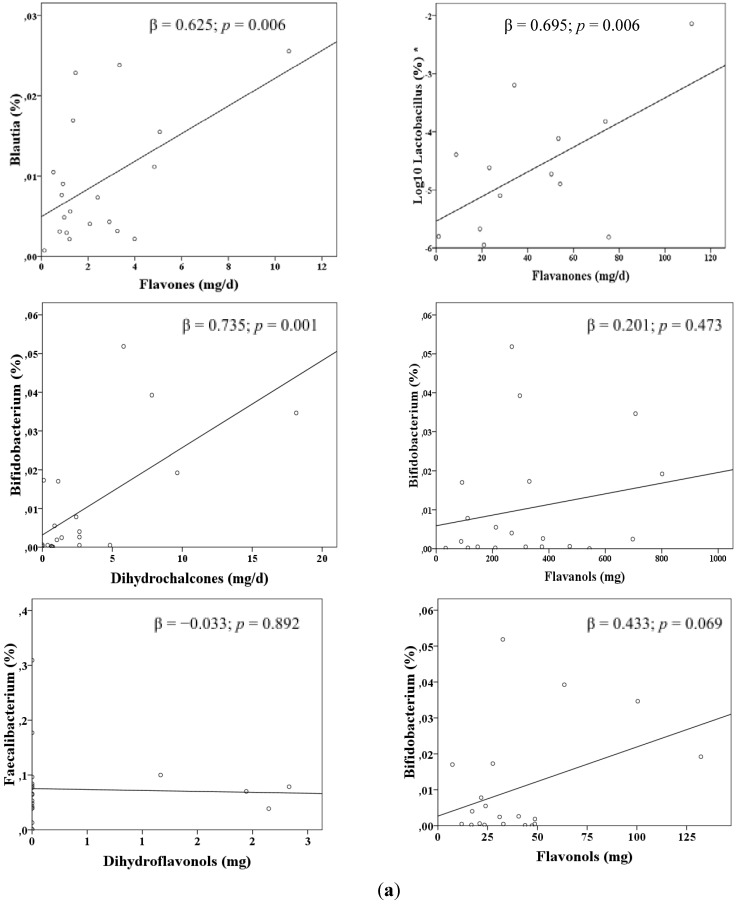

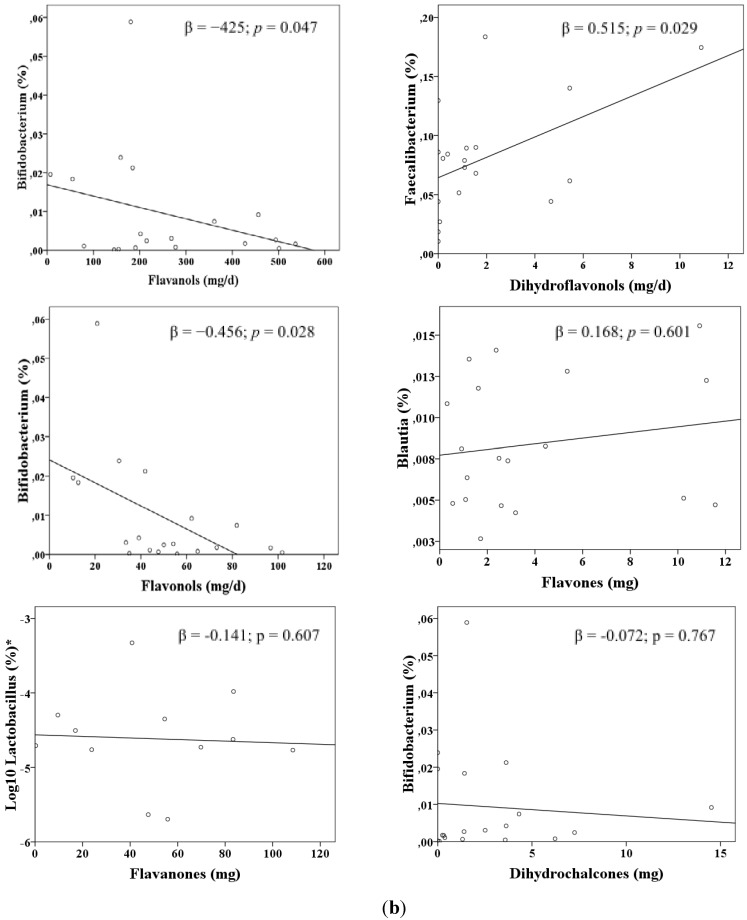
**(a)** Linear estimation trends between the intake of some classes of flavonoids and the proportion of selected microbial genera, in systemic lupus erythematosus patients. Results derived from multiple linear regression analyses including age and energy intake as covariates. **(b)** Linear estimation trends between the intake of some classes of flavonoids and the proportion of selected microbial genera, in controls. Results derived from multiple linear regression analyses including age and energy intake as covariates. * Logarithmically transformed variable.

**Figure 2:**
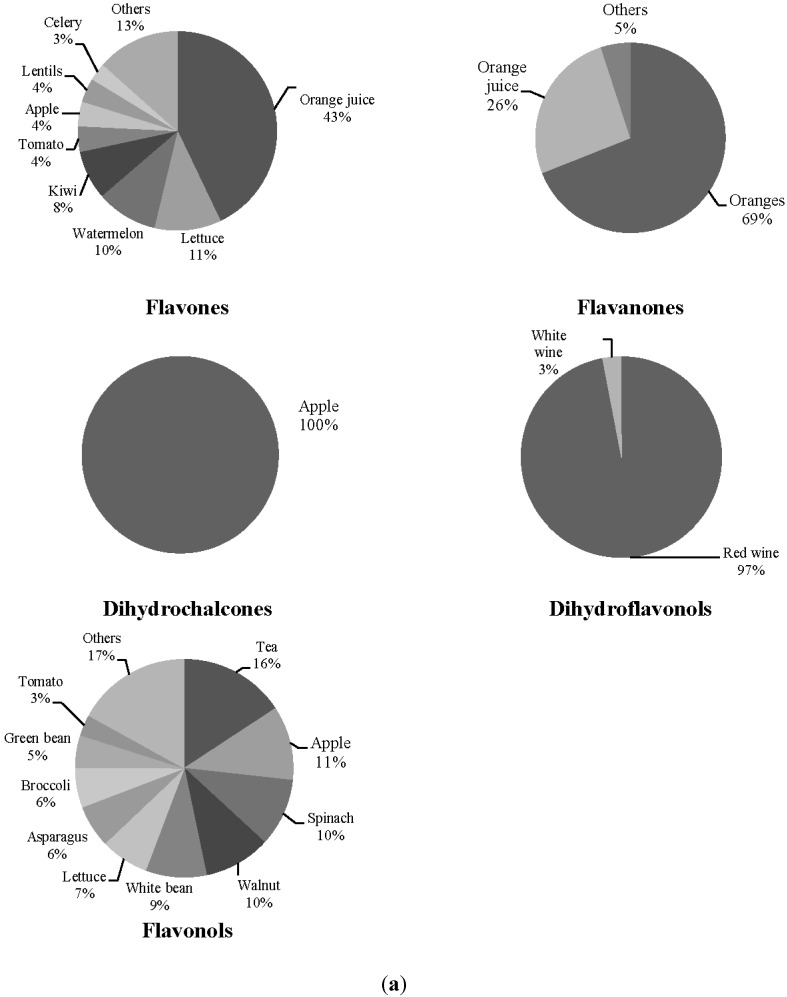

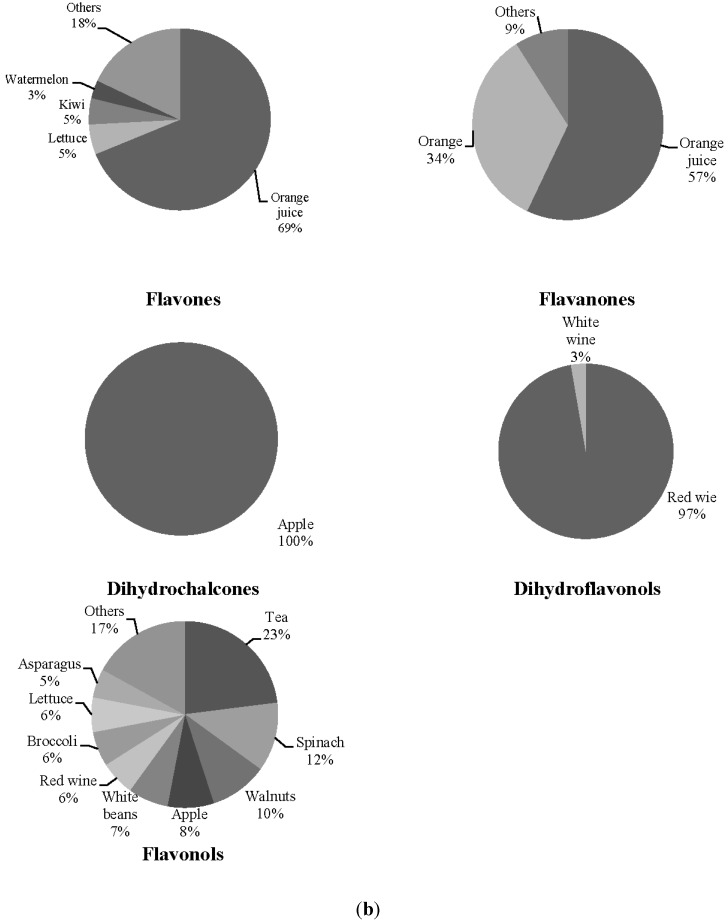
**(a)** Principal food sources of the flavonoid classes previously associated with fecal microbiota, in systemic lupus erythematosus patients. **(b)** Principal food sources of the flavonoid classes previously associated with fecal microbiota, in controls.

The associations found between flavonoid classes and microbiota are illustrated in Figure 1a,b. Results showed positive associations between flavone intake and *Blautia*, flavanones and *Lactobacillus* and dihydrochalcones and *Bifidobacterium* in SLE group. Regarding the control group, dihydroflavonols were directly associated with *Faecalibacterium*, whereas flavanol and flavonol intake were inversely associated with *Bifidobacterium*. Given the high correlation with flavonoids from foods, an additional stepwise regression analysis was conducted to explore the relative importance of flavanol and flavonol intake on *Bifidobacterium*. Flavonol intake was found to be an independent contributor to this microbial group (data not shown). To explore these associations, the main food sources of these flavonoids were calculated (Figure 2a,b). In SLE subjects, oranges and their products were the top contributors to both flavones and flavanones, while dihydrochalcones came exclusively from apple intake. In controls, dihydroflavonols were provided from red wine, and other foods such as tea, spinach and walnuts contributed to explain 45% of flavonol intake.

We did not find differences in the intake of any food sources, with the exception of red wine, which had greater consumption in controls (Table 3).

**Table 3:** Mean intake of the selected food sources of flavonoids in systemic lupus erythematosus (SLE) patients and controls.

	SLE (*N* = 20)	Control (*N* = 20)
Orange (g·day^−1^)	58.43 ± 72.24	34.51 ± 42.75
Orange juice (g·day^−1^)	17.94 ± 33.69	51.51 ± 73.53
Lettuce (g·day^−1^)	50.63 ± 30.19	50.22 ± 42.66
Watermelon (g·day^−1^)	10.79 ± 19.11	7.01 ± 13.83
Kiwi (g·day^−1^)	21.19 ± 29.50	25.11 ± 62.61
Tomato (g·day^−1^)	84.06 ± 51.58	72.77 ± 48.13
Apple (g·day^−1^)	77.81 ± 81.87	58.72 ± 63.59
Lentils (g/day)	8.02 ± 5.12	9.31 ± 7.69
Celery (g·day^−1^)	0.18 ± 0.80	0.45 ± 1.51
Red wine (mL·day^−1^)	6.79 ± 14.37	34.20 ± 52.34 *
White wine (mL·day^−1^)	1.79 ± 7.99	8.95 ± 34.30
Tea (mL·day^−1^)	51.52 ± 156.22	93.82 ± 172.59
Spinach (g·day^−1^)	3.01 ± 6.15	4.44 ± 5.76
Walnuts (g·day^−1^)	6.014 ± 11.24	7.75 ± 10.77
White beans (g·day^−1^)	6.61 ± 4.20	6.82 ± 7.13
Broccoli (g·day^−1^)	6.16 ± 10.63	7.64 ± 9.47
Asparagus (g·day^−1^)	9.66 ± 9.96	8.80 ± 14.87
Green beans (g·day^−1^)	8.08 ± 15.56	11.59 ± 12.36

Derived from Students’ *t*-test; Results are presented as mean ± SD; * *p* ≤ 0.05.

From foods, orange and apple intake explained 38% and 44% of relative abundance for *Lactobacillus* and *Bifidobacterium,* respectively, in SLE subjects, whereas red wine was positively associated with *Faecalibacterium* in the control (Table 4).

**Table 4:** Results from a stepwise multiple regression analysis for prediction of relative abundance of fecal microbiota by the intake of the principal food sources of flavonoids in systemic lupus erythematosus (SLE) and control subjects.

	Predictors	*R*^2^	β	*p*
SLE (*N* = 20)				
*Lactobacillus* ^a^	Orange	0.383	0.619	0.004
*Bifidobacterium* ^b^	Apple	0.437	0.661	0.001
Controls (*N* = 20)				
*Faecalibacterium* ^c^	Red wine	0.264	0.514	0.024

Results derived from multiple stepwise regression analysis; *R*^2^: coefficient of multiple determinations; β: standardized regression coefficient; Variables included in the model: ^a^ age, energy, orange and orange juice intake; ^b^ age, energy, and apple intake; ^c^ age, energy and red and white wine intake. Only significant results are presented.

## 4. Discussion

Systemic lupus erythematosus is a multisystemic chronic inflammatory disease that is autoimmune in nature. Although the cause of this pathology is unknown, accumulating evidence suggests that gut microbiota might impact both disease symptoms and progression. To date, there is no clear evidence about the impact of correcting dysbiosis in these patients, but the identification of dietary factors able to modify the microbial balance could be important for future investigations. With this aim, we proceeded to analyse the association among dietary components and microbiota.

Most previous works about the impact of diet on this disease are focused on the immunomodulatory effects of the supplementation, or restriction of some dietary components, including calorie, protein or fat intake [28,29,30], on the basis that the fact of having a pathological condition could implicate changes in food supply. However, the comparison of the global intake between SLE patients and controls presented in this study reveals that the nutritional intake of both groups is similar and, therefore, there is no dietary constituent significantly raised or lowered in relation to the presence of the pathology.

Despite previous evidence from intervention studies with high-fat diets suggesting that excessive fat intake leads to changes in the proportion of *Bacteroides* and *Clostridium* [31], our results do not support a relationship between fat intake and these bacterial groups in humans with well-balanced diets. Also, we did not find differences in faecal microbiota according to the intake of animal protein or fibre intake. It is possible that the amounts of these dietary components in our sample were insufficient to have an impact on faecal microbiota or, as some authors have suggested, that the food combinations of a diet modify the effect of dietary single components [32]. In this regard, it has been reported that the negative effects of high-fat diets were different when fat was administrated with orange juice *vs.* water or glucose solution [32], probably due to the high levels of antioxidant bioactive compounds, such as flavonoids, present in this beverage, and proposed in recent years as gut microbiota modulators [33].

Likely, the most important finding of this study is the identification of a direct association, in SLE patients, between flavanones, flavones and dihydrochalcones, coming from a regular diet, and faecal levels of *Blautia*, *Lactobacillus and Bifidobacterium*, together with the detection of an inverse association between flavonol intake and faecal levels of *Bifidobacterium*, and positive one for dihydroflavonols and *Faecalibacterium* in control subjects. The positive association between orange flavanones and *Lactobacillus* proportions found in this study is not in accordance with previous evidence of the antimicrobial activity of these flavonoids [34]. Nevertheless, we have limited information about the role of individual flavonoids on microbiota to be able to compare our results, taking into consideration that results from *in vitro* studies cannot be directly extrapolated to what occurs in the physiological context of the intestinal ecosystem, and intervention work often involves very high doses of individual compounds, or high doses of polyphenol rich foods (tea, coffee or cocoa being the most frequent), which are not representative of what occurs in a regular diet.

It is known that the natural presence of flavonoids within foodstuffs and their interaction with other dietary components, such as fibres, may modify the level of polyphenols available to the gut microbiota [35]. In this sense, it is possible that dihydrochalcones from apples, along with dietary fibre, are degraded by *Bifidobacterium*, promoting its growth. This effect of apple polyphenols, previously reported in animal models and humans [36,37], should be of special interest for SLE patients given the immunomodulatory effect attributed to some strains of this bacterial genus [38], such as *B. bifidum* LMG13195, a strain that promoted the induction of regulatory T cells (Tregs), expressing chemokine receptors and potentially favouring mucosal homeostasis [1,2]. Dihydrochalcones have also been shown to influence the commensal intestinal microbiota, increasing the levels of some bacteria in the gut, such as *Lactobacillus* species [39]. As these beneficial groups of microorganisms trigger immunomodulatory processes in the host through the action of metabolites or other secreted compounds, administration of dihydrochalcones might have an impact on host immunity, although this assumption deserves further work [40].

Even though flavones were widely distributed among fruits and vegetables in the sample (oranges, lettuce, watermelon, kiwi, tomato, apple, *etc.*), none of their sources were identified as independent predictors of *Blautia* proportion, so we speculate that the observed associations could be attributable to the combination of all flavone-rich foods present in a whole diet, unlike flavanones and dihydrochalcones, which are provided almost exclusively by oranges and apples, respectively. Since Clostridium-dependent induction of Tregs may be required for maintaining immune homeostasis, and considering that *Blautia* belongs to *Clostridium cluster* XIVa, which promotes Treg cell accumulation, this result could also be of interest for other autoimmune diseases [41].

It is noteworthy that the associations observed in SLE patients did not appear in the controls, which means that in addition to the differences in the food sources of these compounds, it is possible that the variability in the composition of gut microbiota between groups may involve different diet-microbiota associations [42,43], or that subjects with a well-balanced immune system could be less susceptible to the effect of dietary components than subjects with altered immune responses. The influence of red wine on *Faecalibacterium* concentrations has not been previously described, but it is in agreement with the reported changes in the phylum *Firmicutes* after red wine administration [15], supporting the hypothesis about the prebiotic effect of moderate red wine consumption [19]. On the other hand, the inverse association between flavonols and *Bifidobacterium* is in accordance with data from *in vitro* studies [44]. It is possible that the lack of association in SLE patients may be due to the low intake of red wine in this group compared to the controls (5.9 ± 8.9 *vs.* 35.1 ± 9.1 mL·day^−1^ respectively). Since 80% of the SLE sample did not consume red wine, we had no statistical power to detect any association with this beverage.

## 5. Conclusions

Finally, although epidemiological analyses do not establish causality, our findings support the association between polyphenols from a regular diet and faecal microbiota. The association between common foods, such as oranges and apples, with specific microorganisms reported to be decreased in SLE could be of great importance for these patients. It could also be of great interest for future studies to explore the functional changes occurring in SLE intestines and how they affect the various impacts of dietary flavonoids on microbiota for a healthy population. Even when causality between changes in the *Firmicutes/Bacteroidetes* ratio and lupus progression requires further validation, these results will generate new hypotheses to test dietary strategies to correct dysbiosis in this pathology, suggesting a new therapeutic approach to treat autoimmunity diseases.
